# Poor sleep is associated with sensation-seeking and risk behavior in college students

**DOI:** 10.5935/1984-0063.20220024

**Published:** 2022

**Authors:** Laila Taghvaee, Amir Ali Mazandarani

**Affiliations:** 1 Islamic Azad University, Psychology - Tehran - Tehran - Iran.; 2 The Institute for Research and Development in the Humanities (SAMT), Behavioral Sciences - Tehran - Tehran - Iran.

**Keywords:** Risk-Taking, Sleep Deprivation, Sensation

## Abstract

**Objective:**

The current study examines the relationship between sleep duration and variability with sensation- seeking and risky behaviors in a sample of Iranian undergraduate students.

**Methods:**

The participants in this study were 385 undergraduate students in Tehran. To collect data, we used the Sensation Seeking Scale, Risk Behavior Scale, and two questions related to sleep duration and sleep variability.

**Results:**

The results showed a positive relationship between sleep deprivation and irregular sleep schedule with sensation-seeking and risk behavior tendencies in students.

**Discussion:**

This association could point to an indirect impact of sleep deprivation on sensationseeking and risk behavior, for example, by altering brain regions responsible for self- control.

## INTRODUCTION

Sensation-seeking and risk-taking propensities are prevalent in young adulthood ^1^ and may have lifelong negative consequences. Sensation seeking is associated with alcohol use ^2^ and aggression ^3^, and risky behaviors may lead to motor vehicle crashes, homicide, and suicide, which are the leading causes of death for all young people aged 18 to 24 years ^4^. It seems that one of the factors affecting sensation-seeking tendencies and risk-taking behaviors is sleep problems.

Sleep deprivation refers to having less than the recommended amount of sleep, which for adults is between seven and nine hours a night ^5^. Sleep deprivation is a severe and rising issue in modern society, especially among university students, and the number of students who are routinely sleep-deprived due to increasing psycho-social stresses. Most college students are sleep deprived, with 70.6 percent reporting getting less than 8 hours of sleep a night. Their sleep schedule is also irregular. They tend to delay both bedtimes and rise times at weekends. Furthermore, university students suffer from poor sleep quality ^6,7^. Due to the negative impacts of insufficient and irregular sleep schedules on physical and mental health, the widespread prevalence of sleep problems among students can be considered a significant public health problem.

Sensation seeking is a personality trait characterized by a generalized preference for diverse, novel, complex, and intense sensations and experiences, as well as a desire to take risks to achieve those experiences ^8^. There has been limited research on the link between poor sleep and sensation-seeking predisposition. For example, Killgore ^9^ demonstrated that one night of sleep deprivation *decreased* self-reported sensation-seeking significantly. He explained his observation by claiming that sleep deprivation is associated with significant physical and mental exhaustion, as well as a sense of diminished energy and vigor, and the majority of the items on the Sensation-Seeking Scale necessitate some level of energy expenditure. in other studies ^10,11^, high sensationseeking was unrelated to insomnia severity and sleep length.

In addition, high levels of sensation-seeking may have advantages. High sensation seekers, for example, respond better to external stressors like violence and captivity than low sensation seekers ^12^. As a result, they also could be more resistant to the stress of poor sleep. In a recent study, McGowan and her colleagues 13 found that in comparison to days after average and poor sleep quality, momentary sensation-seeking peaks higher and earlier after nights with better sleep quality. They concluded that sleep quality could help people search out new experiences in everyday life without rash decisions.

In converse, in a study by Rusnac, Spitzenstetter, and Tassi ^14^, sleep deprivation was associated with increased sensation-seeking, mainly when the sleep deprivation was voluntary. In addition, in several studies, university students with an evening circadian preference score higher than those with a morning preference on the Sensation-Seeking ^15^, and when compared to morning types, evening types have more inferior sleep quality and shorter sleep length ^16^. High levels of sensation-seeking are also linked to various adverse outcomes, including higher levels of risk-taking ^17^.

Sleep loss, both chronic and acute, is related to increased reward sensitivity and risk-taking in adults ^18^ and adolescents ^19^. According to a large-scale analysis of military service members ^20^, longer sleep time decreases the likelihood of risk-taking behaviors and predicts a lower chance of committing any high-risk behavior. The effect of sleep loss on increased risk-taking may be due to several underlying cognitive mechanisms, including inattention or attentional lapses, lack of awareness of actual sleepiness and impairments caused by it, and insufficient inhibitory control. Therefore, sleep loss can also increase risk by reducing inhibitory capacity. Furthermore, when gains are emphasized, sleep deprivation leads to a greater willingness to take risks. ^21^.

Sleep deprivation can alter behavioral, cognitive, and emotional factors that increase risk-taking ^22^. Sleep loss temporarily alters brain function, especially in areas in the prefrontal cortex, most responsible for self-regulation. According to preliminary research ^23^, the neurophysiological changes caused by prolonged sleep deprivation may even trigger novel manic-psychotic episodes. Some of these changes can affect cognitive functions, enhancing the propensity to participate in risky behaviors. Sleep deprivation has been attributed to impulsivity as well as alterations in reward-related decision-making ^24^. According to a recent study, five nights of partial sleep deprivation decreased data collection before making decisions and increased risk-taking more than after one night of complete sleep deprivation ^25^. Since many students in Iran are chronically and partially sleep deprived ^7^, this result has unique implications for public health policy and practice in Iran.

Research to date has often shown conflicting findings on the complex relationship between sleep indices with sensation-seeking and risk-taking propensities. In one study ^26^, poor sleep was linked to more reckless driving. However, good sleep was protection against risky driving in adolescents who had less sensation-seeking tendencies. Therefore, Sensation-seeking may moderate the relation between poor sleep and risk behavior. Data from a study of adolescents and young adults ^27^ showed that sleep duration and delayed bedtimes are associated with delinquent behavior. These effects are mediated by sensation-seeking and impulse control. Similarly, research ^28^ has shown that regardless of sleep adequacy, sensation-seeking is associated with higher alcohol intake by young adults. In contrast, premeditation is only protective against binge drinking in people who get sufficient sleep.

Most research on the connection between sleep and sensation-seeking and high-risk behaviors has concentrated on sleep deprivation, with little attention given to sleep schedule variability. The present research, however, aims to investigate the relationship between both sleep length and variability with sensation-seeking and risk behavior. We hypothesize that both partial sleep deprivation and irregular sleep schedule would be associated with sensation-seeking and risk behavior.

## MATERIAL AND METHODS

We used a descriptive cross-sectional approach to assess the association between sleep deprivation and irregular sleep with sensation-seeking and risk behaviors in students. The study was reviewed and approved by the ethics committee of Soore University in Tehran.

### Participants

During Stay-at-Home orders and since university classes were only held online due to the COVID-19 outbreak, a link to a form containing the study instruments was sent to major student social media groups at three universities in Tehran between July 13, 2020, and January 10, 2021. Overall, the forms were entirely completed by 385 undergraduate students. We designed the form so that responses to all items were mandatory, so there was no missing data or an incomplete form. The students’ ages ranged from 18 to 30 years (M = 23.37; SD = 3.7; 68.6 % were female; and 82.3 % were single).

### Statistical Procedures

For statistical procedures, we used IBM SPSS 26. Depending on the types of variables, we calculated Pearson, biserial, and phi correlation coefficients and then assessed their statistical significance. For between-group comparisons, we used several two-way analyses of variances to determine the interactive effect of sleep duration and variation. We conducted many statistical tests, therefore, we needed to control for multiple testing. All P-values were adjusted based on Benjamini-Hochberg’s correction for decreasing the false discovery rate ^29^. To do this, we used a syntax available on the IBM website ^30^. To determine effect sizes, partial eta-squared was used, with small, medium, and large effects defined as .01, .06, and .14 or higher, respectively ^31^.

### Research Instruments

#### Sensation Seeking Scale Form-V^32^

To assess individual differences in trait sensationseeking, we chose the Persian version of Zuckerman’s SSS, adapted by Mahvi-Shirazi ^33^. The SSS consists of 40 items and four subscales with ten items each: disinhibition (DIS), boredom susceptibility (BS), thrill and adventure-seeking

(TAS), and experience seeking (ES). We asked participants to choose one of two alternative descriptions for each item. Based on their choices, items were coded as 0 or 1 and then summed to obtain the total SSS score and the four scale scores. The reliability and validity of SSS are confirmed in different countries ^34,35^. In the present study, the overall SSS score and three out of the four subscales showed satisfactory internal consistency (a = .97 for DIS; a = .66 for BS; a = .77 for TAS; a = .92 for ES, and a = .92 for overall SSS).

#### Risk Behaviors Scale^36^

We assessed high-risk behaviors using the Risk Behaviors Scale. The scale’s items explore the prevalence of high-risk behaviors in the context of Iran’s cultural and social constraints. This questionnaire contained 61 items, with responses ranging from 0 to 4 on a 5-point Likert scale. According to Shafiee ^36^, experts and professors supported the content validity of the scale. To assess construct validity in this research, we used exploratory factor analysis with the Principal Component approach and Varimax rotation. Following factor analysis, we omitted 16 poor items (items with cross-loading or loading less than 0.5). After we removed the unreliable items, The Kaiser-Mayer-Olkin coefficient (.97) was excellent, and the Bartlett test of sphericity coefficient was X^2^_approx_ = 17,733.20, P<0.0001, implying adequate factors can be derived from the data. The second exploratory factor analysis with the same method on the remaining 45 items resulted in a five factors solution with eigenvalues greater than 1 (a = .970 for overall scale): Violence, drug use, and HIV risk (19 items; a = .97), Tobacco and Alcohol Experiences (10 items; a =.95), anger and family conflicts (8 items; a = .92), Unhealthy diet (6 items; a = .87), and overweight and dieting (2 items; a =0.75). These five factors explained 51.47% of the total variance in the data. For more details about the Risk Behavior Scale and its factors, items, and loadings, see the Appendix at the end of the current article. In the present study, the participants’ overall score on this scale positively correlated with their overall score on the SSS (Pearson’s correlation coefficient = 0.7).

#### Sleep Duration and Variability

Sleep duration and variability were measured by two single items: “*How many hours of sleep per 24 hours do you normally get?*” In the present study, in line with many other studies ^37^, laboratory studies ^38^, and public opinion ^39^, the criterion for partial sleep deprivation was considered as sleeping less than 6 hours a day. We split participants into two groups: those who typically sleep 6 hours or more and those who sleep less than 6 hours a day. In several studies ^27^, researchers have used a similar question to measure sleep duration. Also, sleep variability was measured by a single item asking students to rate their agreement with the statement, “*My sleep schedule is irregular*” by choosing “*yes*” or “*no*”. There is still no clear definition of sleep variability, nor is there any research-based recommendation to determine how much variation in sleep timing is desirable ^40^. Therefore, we provided this definition in the questionnaire so that participants would understand what we meant by irregular sleep schedule: “By sleep irregularity, we imply a difference of more than two hours between bedtimes and/or rising-times on weekdays and weekends.” Research ^41,42^ has confirmed a moderate concurrent validity of these kinds of self-reported subjective measures by showing high agreement between subjective and objective measures of sleep.

## RESULTS

Only 73 students (19%) reported that, on average, they slept less than 6 hours a night. However, 223 participants (57.9%) reported having irregular sleep patterns during the weekdays.

### Correlations

Table 1, shows the means, standard deviations, and correlations between the research variables. Sleep deprivation and variability were positively correlated to sensation-seeking and risk behavior and all but one of their sub-scales (P < .01). However, all the coefficient values were moderate to low. Specifically, reported sleep deprivation was moderately correlated with sensation seeking (r = .39; P < .001), and slightly correlated with risk behavior scores (r = .26; P < .001). Also, reported sleep variability was moderately correlated with sensation seeking (r = .49; P < .001) and risk behavior scores (r = .39; P < .001).

**Table 1.** Means, standard deviations, and correlations between all variables (n = 385).

	M	SD	1	2	3	4	5	6	7	8	9	10	11	12
1. Disinhibition	4.25	2.28												
2. Boredom Susceptibility	3.94	2.84	.66**											
3. Thrill and Adventure Seeking	5.00	3.75	.68**	.65**										
4. Experience Seeking	4.27	2.66	.75**	.78**	.76**									
5. Sensation Seeking	14.91	8.70	.83**	.86**	.91**	.90**								
6. Violence, Drug use, and HIV Risk	7.99	13.70	.58**	.62**	.48**	.62**	.59**						
7. Tobacco and Alcohol Experiences	10.89	10.77	.67**	.62**	.52**	.65**	.66**	.76**						
8. Anger and family conflicts	9.59	7.33	.65**	.54**	.55**	.66**	.64**	.69**	.71**					
9. Unhealthy diet	9.49	5.22	.53**	.45**	.46**	.53**	.53**	.55**	.62**	.67**				
10. Overweight and dieting	1.31	1.83	.26**	.10	.21**	.19**	.20**	.15*	.22**	.34**	.31**			
11. Risk behavior	39.27	33.10	.70**	.66**	.58**	.71**	.70**	.91**	.91**	.86**	.75**	.32**		
12. Sleep Deprivation	1.19	.39	.37**	.36**	.32**	.38**	.39**	.24**	.23**	.22**	.15*	.14*	.26**	
13. Sleep Variability	1.58	.49	.45**	.38**	.47**	.44**	.49**	.29**	.34**	.41**	.36**	.21**	.39**	.21**

Sleep Deprivation and sleep Variability are dichotomous variables (i.e., no=1, yes =2); as a result, we calculated the correlation between them with the phi correlation coefficient, and we calculated their correlation with other variables by the biserial correlation. For other variables, we used the Pearson correlation coefficient. P-values are adjusted using Benjamini-Hochberg’s method; * P < .01; ** P < .001.

### Between-group comparisons

We conducted 11 two-way ANOVAs to examine the combined effects of sleep duration (less than 6 hours vs. 6 hours and more) and sleep variability (irregular vs. regular sleep schedule) on sensation seeking and risk behavior and their subscales. As shown in Table 2, The interactive effect of sleep duration and sleep variability on overall sensation-seeking was significant (P < .05; partial ?^2^ = .016). The interactive effect was also significant for disinhibition subscale (P < .05; partial ?^2^ = .016). The interactive effect of sleep duration and sleep variability on overall risk behavior was not significant. However, the combined effect on Anger and family conflicts was significant (P < .05; partial ?^2^ = .012).

**Table 2:** Two-way ANOVA results on the influence of sleep duration, sleep variability, and interaction between sleep duration and sleep variability on sensation seeking and risk behavior and their subscales.

	Scales Parameters	Good-Sleepers M (SD)^a^	Poor-Sleepers M (SD)^b^	F^C^	Adjusted *P*-Values ^C^	partial eta^2C^
Disinhibition	Sleep Duration	3.84 (2.16)	6.01 (1.90)	48.35	<.01	.113
	Sleep Variability	3.05 (1.75)	5.13 (2.22)	17.74	<.01	.044
	Interaction	-	-	6.08	<.05	.016
Boredom Susceptibility	Sleep Duration	3.45 (2.53)	6.03 (3.13)	39.11	<.01	.093
	Sleep Variability	2.66 (2.14)	4.87 (2.92)	12.50	<.01	.032
	Interaction	-	-	2.95	>.05	.008
Thrill and Adventure Seeking	Sleep Duration	4.42 (3.68)	7.52 (2.91)	31.22	<.01	.076
	Sleep Variability	2.92 (3.40)	6.52 (3.23)	25.36	<.01	.062
	Interaction	-	-	4.06	>.05	.011
Experience Seeking	Sleep Duration	3.78 (2.55)	6.36 (2.07)	46.17	<.01	.108
	Sleep Variability	2.89 (2.03)	5.26 (2.63)	19.18	<.01	.048
	Interaction	-	-	3.64	>.05	.009
Sensation Seeking	Sleep Duration	13.26 (8.27)	21.95 (8.70)	53.81	<.01	.124
	Sleep Variability	9.88 (7.39)	18.57 (7.70)	25.71	<.01	.063
	Interaction	-	-	6.02	<.05	.016
Violence, Drug Use, and HIV Risk	Sleep Duration	6.39 (12.11)	14.82 (17.56)	12.30	<.01	.031
	Sleep Variability	3.24 (5.75)	11.43 (16.50)	10.21	<.01	.026
	Interaction	-	-	.213	>.05	.001
Tobacco and Alcohol Experiences	Sleep Duration	9.67 (10.0.3)	16.12 (12.25)	10.19	<.01	.026
	Sleep Variability	6.63 (7.50)	13.99 (11.70)	15.86	<.01	.040
	Interaction	-	-	.106	>.05	<.001
Anger and family conflicts	Sleep Duration	8.83 (7.23)	12.86 (6.88)	12.69	<.01	.032
	Sleep Variability	6.04 (5.00)	12.17 (7.67)	16.04	<.01	.040
	Interaction	-	-	4.52	<.05	.012
Unhealthy diet	Sleep Duration	9.11 (5.30)	11.10 (4.61)	7.56	<.05	.019
	Sleep Variability	7.29 (4.62)	11.09 (5.07)	8.10	<.01	.021
	Interaction	-	-	7.21	<.05	.019
Overweight and dieting	Sleep Duration	1.19 (1.75)	1.85 (2.05)	5.39	<.05	.014
	Sleep Variability	.86 (1.45)	1.64 (2.00)	2.77	>.05	.007
	Interaction	-	-	1.29	>.05	.003
Risk behavior	Sleep Duration	35.18 (30.79)	56.75 (36.97)	15.55	<.01	.039
	Sleep Variability	24.07 (19.68)	50.31 (36.38)	17.20	<.01	.043
	Interaction	-	-	1.63	>.05	.004

a. For the “sleep duration” factor, “good sleepers” refers to participants who reported at least 6 hours of sleep a night (N = 312); for the “sleep variability” factor, it refers to participants who reported a regular sleep schedule (N = 162); b. For the “sleep duration” factor, “poor sleepers” refers to participants who reported less than 6 hours of sleep a night (N=73); for the “sleep variability” factor, it refers to participants who reported irregular sleep schedule (N=223); c. Reported Fs, adjusted P-values, and effect sizes are based on the main effects in the two-way ANOVA model. P-values are adjusted using Benjamini-Hochberg’s method.

According to the principle of marginality, we do not report the main effects in the presence of interaction effects. The main effects of sleep duration on the other three sensation-seeking subscales (i.e. Boredom Susceptibility, Thrill and Adventure Seeking, and Experience Seeking) were significant (Ps < .01; partial ?^2^s = .093, .076, and .108 respectively). In other words, students who reported sleeping less than 6 hours a night scored higher on the three sensation-seeking sub-scales. The main effect of sleep duration on overall risk behavior score was also significant (P < .01; partial ?2 = .039). Finally, the main effects of sleep duration on the other four risk-behavior subscales (i.e. Violence, Drug Use, and HIV Risk, Tobacco and Alcohol Experiences, Unhealthy diet, and Overweight and dieting) were significant (Ps for first two < .01 and the second two < .05; partial ?^2^s = .031, .026, .019 and .014 respectively). In other words, students who reported sleeping less than 6 hours a night scored higher on the risk behavior scale and the four sub-scales mentioned.

In addition, the main effects of sleep variability on the three sensation-seeking subscales (i.e. Boredom Susceptibility, Thrill and Adventure Seeking, and Experience Seeking) were significant (Ps < .01; partial ?^2^s = .032, .062, and .048 respectively). That is to say, students who reported sleeping on an irregular schedule scored higher on the three sensation-seeking sub-scales. The main effect of sleep variability on overall risk behavior score was also significant (P < .01; partial ?2 = .043). That means students who reported irregular sleep patterns, also reported more risky behaviors. Finally, the main effects of sleep variability on three subscales of the risk-behavior scale (i.e. Violence, Drug Use, and HIV Risk, Tobacco and Alcohol Experiences, and Unhealthy diet) but not for the *overweight and dieting* subscale were significant (Ps < .01; partial ?^2^s = .026, .040, and .021 respectively). In other words, students who reported more variable sleep patterns scored higher on the risk behavior scale and the three sub-scales.

## DISCUSSION

The results showed that an insufficient and, or irregular sleep schedule was associated with risk behavior and sensation-seeking tendencies. Interestingly, compared to previous research ^6,7^, a small percentage of students reported insufficient sleep, while most of them reported variable sleep schedules. This observation is most likely due to conditions brought on by the Covid-19 epidemic. Students were likely to have more chances to sleep at home during the Covid-19 pandemic, but their sleep schedules were disturbed. This hypothesis is consistent with the previous study ^43^, indicating an improvement in sleep duration and delayed sleep timing among students during the Covid-19 pandemic.

One of the main findings of this study was the association between irregular sleep schedules with sensationseeking and risk behavior propensities in undergraduate students. To the best of our knowledge, no research has been done on the relationship between sleep variability with sensation-seeking and risk behavior tendencies in young adults. Inconsistent sleep schedule has been shown to increase the risk of problem behavior in school-aged children ^44^. While Troxel et al. ^37^ discovered that adolescents with a more consistent sleep pattern engage in *more* risky sexual behaviors than those with a more variable schedule, they also stated that lack of variability might indicate chronic inadequate sleep on both weekdays and weekends. According to one study ^45^, sleep variability in adolescents, regardless of duration, decreases brain development in many regions, some of which are essential for behavioral inhibition and self-regulation.

The findings may be explained using Baumeister’s self-regulation theory and the concept of self-depletion ^46^. Self-control, according to the theory, requires purposeful effort sustained by cognitive resources depleted by the act of exercising focused and persistent self-control. Ego depletion is a temporary reduction in one’s ability or willingness to participate in volitional activities. Sleep, according to Baumeister, Wright, and Carreon ^47^, is critical for restoring the depleted cognitive resources. According to their findings, reports of ego depletion spiked following poor sleep quality and quantity. Barber and her colleagues ^48,49^ proposed that sleep consistency and sufficiency are needed for improved self-regulation. They hypothesized that in addition to the restorative advantages of adequate sleep length, long-term regular sleep habits might help people develop self-regulatory capability by requiring them to exercise self-control. In a study of adolescents ^50^, researchers related sleep deprivation to poor self-control, while they linked poor self-control to delinquency. In the present study, we propose that insufficient and irregular sleep increases students’ sensation-seeking and risk-taking tendencies by causing self-depletion and lowering self-control.

We must interpret our results in light of the study’s limitations, such as the single-item self-reported measurements of sleep length and variability of sleep schedule. Although some studies have confirmed the validity of subjective self-reported sleep ^41,42^, others ^51^ showed poor agreement between subjective and objective sleep measures. As a result, we cannot be certain if the people who reported inadequate sleep were sleep-deprived. However, regardless of how precise these items are at measuring sleep indices, we argue that they can display the *perceived* length and variability of sleep. Since it is linked to essential daytime functions ^52^, sleep perception can be as crucial as actual sleep.

The difficulty of defining the exact nature of the interactions between variables is a second limitation. The findings may also be interpreted in the opposite direction: people with high scores in sensation-seeking and risk-taking may choose short sleep duration or irregular sleep patterns. As a result, the correlational nature of this study restricts its interpretation in terms of the direction of the relationship. Sleep deprivation and variability were found to be associated with increased sensation-seeking and risk-taking behaviors. Although this is in line with theoretical assumptions, we are unable to pinpoint the precise neurological or psychological mechanisms that link the two. More research is needed to determine the relative contributions of other potentially confounding physiological (e.g., functioning of prefrontal regions such as the dorsolateral prefrontal cortex), individual (e.g., personality or lifestyle), and social (e.g., social support or socioeconomic status) factors that may underpin both poor sleep and these risk-seeking tendencies.

Our results show that sleep variability and sufficiency are associated with sensation-seeking and risk behavior tendencies and their subscales. Students who have irregular and inadequate sleep have higher scores on sensation-seeking and risk-taking indicators. They are more inclined to engage in violence, rage, and family disputes. They have a higher risk of being involved in a traffic accident, use drugs, and alcohol, and are more likely to get infected with HIV. These findings, if repeated using accurate measurements, could shed light on the connection between sleep, sensation-seeking, and risk-taking tendencies.

## Appendix

**Table:** Appendix: Risk Behavior Scale: Factors, Items, Eigenvalues, Means (SD), and Loadings.

Factors and Items	Eigenvalues	M (SD)	Load
**1. Violence, Drug Use, and AIDS**	**23.16**	**7.99 (13.70)**	
I have taken drugs with my friends at private parties		.32 (.88)	.89
For self-defense, I often carry a knife, brass knuckles, a blade cutter, or other weapons		.33 (.86)	.84
I often use other people's toothbrushes		.13 (.41)	.83
I have tried snuff and Naswar		.32 (.88)	.82
I sometimes take pills and smoke with my friends because I don’t want to chicken out		.39 (.93)	.81
I have a special group with my friends for fights.		.43 (.97)	.79
I have participated in several group fights with my friends.		.51 (1.04)	.79
It is normal to use drugs in our house.		.17 (.57)	.79
I usually like group physical combats.		.44 (.92)	.78
At the gym, I used a shared needle to inject vitamins and supplements		.17 (.56)	.75
In situations, I smoked with my friend to not seem a coward to them		.47 (1.00)	.75
I have touched the blood of others several times (in fights, injuries, accidents, etc.)		.36 (.80)	.75
I have self-harmed several times in physical combats		.35 (.85)	.72
I am known as an aggressive person		.65 (1.00)	.72
My friends take drugs or pills to relieve depression and mood swings.		.52 (.96)	.68
In some situations, I have used the razor blade of others		.30 (.70)	.67
People more often get angry with me than with others		.65 (.95)	.60
I often intimidate my younger siblings angrily		.84 (1.01)	.59
My friends often smoke and use snuff at university or work		.62 (1.03)	.57
**2. Tobacco and Alcohol Experiences**	**3.63**	**10.89 (10.77)**	
I have tried alcoholic drinks		.97 (1.30)	.75
My friends use alcoholic drinks in private		1.51 (1.42)	.75
My friends smoke hookah in recreational places.		1.74 (1.38)	.70
My friends have offered me a cigarette		1.03 (1.25)	.69
I have tried hookah with fruit flavor		1.54 (1.38)	.68
I have been offered alcoholic drinks at wedding parties		.95 (1.15)	.67
I have tried cigarette smoking a few puffs		.90 (1.28)	.66
I have tried alcoholic drinks that made me lose myself		.61 (1.11)	.65
I have tried smoking a whole cigarette		.88 (1.38)	.65
People around me have been stoned by drugs		.77 (1.14)	.56
**3. Anger and family conflicts**	**1.98**	**9.59 (7.33)**	
At home, the irrational behaviors of my parents drive me crazy		1.40 (1.29)	.75
I am fed up with the harsh treatment and conflict with my family		1.21 (1.18)	.71
I have had several suicidal thoughts in the past year		.51 (.95)	.70
I have verbal conflicts with my family members		1.29 (1.08)	.66
My parents are strict with me.		1.33 (1.21)	.61
Sometimes I feel like exploding with anger like a barrel of gunpowder		1.32 (1.16)	.58
It is difficult for me to follow the rules of our house		1.21 (1.12)	.56
When I get angry, I have no control over what I say.		1.33 (1.16)	.55
**4. Unhealthy diet**	**1.85**	**9.49 (5.23)**	
I often eat pizza and sandwiches.		1.75 (1.01)	.80
I always enjoy eating potato chips and chocolate as a snack.		2.01 (1.15)	.73
I like fatty foods.		1.63 (1.12)	.71
I do not limit the consumption of fats, sweets, and condiments		1.63 (1.16)	.69
I like salty foods.		1.42 (1.14)	.59
People around me believe that I eat unhealthy foods.		1.05 (1.11)	.51
**5. Overweight and dieting**	**1.37**	**1.31 (1.82)**	
I take strict diets to lose weight.		.55 (.84)	.87
I feel fat compared to my friends.		.77 (1.18)	.85
